# Response to Letter to the Editor: “Prevention of Adrenal Crisis: Cortisol Response to Major Stress Compared to Stress Dose Hydrocortisone Delivery”

**DOI:** 10.1210/clinem/dgaa712

**Published:** 2020-10-08

**Authors:** Alessandro Prete, Angela E Taylor, Irina Bancos, David J Smith, Mark A Foster, Sibylle Kohler, Violet Fazal-Sanderson, John Komninos, Donna M O’Neil, Dimitra A Vassiliadi, Christopher J Mowatt, Radu Mihai, Joanne L Fallowfield, Djillali Annane, Janet M Lord, Brian G Keevil, John A H Wass, Niki Karavitaki, Wiebke Arlt

**Affiliations:** 1 Institute of Metabolism and Systems Research, University of Birmingham, Birmingham, UK; 2 Centre for Endocrinology, Diabetes and Metabolism, Birmingham Health Partners, Birmingham, UK; 3 Division of Endocrinology, Metabolism and Nutrition, Department of Internal Medicine, Mayo Clinic, Rochester, MN, USA; 4 School of Mathematics, University of Birmingham, Birmingham, UK; 5 Institute of Inflammation and Ageing, University of Birmingham, Birmingham, UK; 6 NIHR Surgical Reconstruction and Microbiology Research Centre, Queen Elizabeth Hospital, Birmingham, UK; 7 Royal Centre for Defence Medicine, Queen Elizabeth Hospital, Birmingham, UK; 8 Oxford Centre for Diabetes, Endocrinology and Metabolism, Churchill Hospital, Oxford, UK; 9 Department of Endocrinology, Diabetes and Metabolism, Evangelismos Hospital, Athens, Greece; 10 Department of Anaesthesiology, Royal Shrewsbury Hospital, The Shrewsbury and Telford Hospital NHS Trust, Shrewsbury, UK; 11 Department of Endocrine Surgery, Churchill Hospital, Oxford, UK; 12 Institute of Naval Medicine, Alverstoke, UK; 13 Critical Care Department, Hôpital Raymond-Poincaré, Laboratory of Infection & Inflammation U1173 INSERM/University Paris Saclay-UVSQ, Garches, France; 14 Department of Clinical Biochemistry, University Hospital of South Manchester, Manchester Academic Health Science Centre, The University of Manchester, Manchester, UK; 15 NIHR Birmingham Biomedical Research Centre, University of Birmingham and University Hospitals Birmingham NHS Foundation Trust, Birmingham, UK

Our study (1) included a simple 1-compartment pharmacokinetic model to interpret the results of stress dose hydrocortisone administration in patients with adrenal insufficiency and to predict the outcome of a modified protocol to treat these patients during major stress. The model was fitted to hydrocortisone IV bolus data, then used to retrodict responses to continuous hydrocortisone IV infusion and predict dynamic responses to the combination of the two, including both the initial transient as well as the eventual steady state.

Dorin et al. (2) highlight that our 1-compartment model may underpredict the rate of cortisol rise during continuous infusion. They recommend instead the use of a 3-compartment model (e.g., that of Picard-Hagen et al. (3)), accounting for both free and bound compartments for cortisol, in addition to the peripheral compartment.

We used a simplified model because we had only data available that combined free and bound circulating cortisol, which makes it challenging, if not impossible, to constrain the parameters of a 3-compartment model. However, Dorin et al. (2) are certainly correct that the 1-compartment model is insufficiently complex to capture the kinetics of both bolus and continuous IV data simultaneously. Therefore, we have implemented a model along the lines they suggested and evaluated the robustness of our prediction.

In brief, our 1-compartment ordinary differential equation model (1) for total cortisol *c* took the form,


$$\begin{array}{l} \frac{dc}{dt}=-kc+q \end{array}$$ (1)

A 3-compartment model (2), when rewritten in notation consistent with our paper, divides cortisol into free (*c*_*f*_), bound (*c*_*b*_), and peripheral compartment *c*_*p*_, with only free cortisol undergoing removal:


$$\begin{array}{l} \begin{array}{ll} & \frac{dc_{f}}{dt}=q\left(t\right)-k_{ex}c_{f}-k_{fb}c_{f}b+k_{bf}c_{b}-k_{fp}c_{f}+k_{pf}c_{p},\\ & \frac{dc_{b}}{dt}=k_{fb}c_{f}b-k_{bf}c_{b},\\ & \frac{dc_{p}}{dt}=k_{fp}c_{f}-k_{pf}c_{p},\\ & \frac{db}{dt}=-k_{fb}c_{f}b+k_{bf}c_{b}, \end{array} \end{array}$$ (2)

where *b* is a variable representing the reserve capacity of a binding compartment consisting of albumin and corticosteroid-binding globulin (CBG). A related 3-compartment model that does not include a peripheral compartment but does account for albumin and CBG separately was described by Dorin et al. (4); we expect the similar model structure would produce similar results.

The 3-compartment model (2) contains 7 free parameters, a dilution constant (absorbed into the function *q*(*t*)), removal rate *k*_*ex*_, binding rate *k*_*fb*_, unbinding rate *k*_*bf*_, transport to and from peripheral compartment *k*_*fp*_, *k*_*pf*_, and maximum binding compartment capacity *b*_0_.

As noted by Picard-Hagen et al. (3), the binding/unbinding rates *k*_*fb*_ and *k*_*bf*_ are fast, which led them to simplify the model via quasi-steady kinetics. For brevity, we instead note that the results will be relatively insensitive to both the absolute values of these rates and their ratio, and set *k*_*bf*_ = 1 min^–1^ and *k*_*fb*_ = 0.1 L nmol^–1^ min^–1^ (e.g., we find changing their ratio by a factor of 10 leads to less than 1% improvement in the log-likelihood). Neither parameter value should be taken at face value; the point is that changing them will not significantly change the model predictions.

Based on the parameter estimates of Picard-Hagen et al. (3), we will take the rate of transport to periphery to be similar to the rate of removal, specifically *k*_*fp*_ = *k*_*ex*_, and rate of return from periphery approximately half of this, specifically *k*_*pf*_ = 0.5*k*_*fp*_. Based on these assumptions, the 3-compartment model then has only 3 remaining free parameters: α, *k*_*ex*_, and *b*_0_. Simultaneous maximum likelihood fitting of the model shows a reasonable fit to both bolus and continuous IV data, giving greater confidence in its predictions for the combined treatment. A caveat is that the continuous IV data show some evidence of a gradual upregulation of clearance over the 24-hour span, which is not included in the model.

However, the combined treatment predictions based on the 3-compartment model (2) show very few differences to our 1-compartment model (1), with only modification of the very early time dynamics, showing very high concentrations immediately after the bolus, followed by a faster equilibration than predicted in our paper (1). Our core prediction is, however, confirmed and reinforced: a 50-mg IV bolus of hydrocortisone followed by continuous IV infusion of 200 mg/24 h is optimal to achieve rapid and consistent stress-like response.

## Additional Information

***Disclosure Summary:*** All authors declare: no support from any organization for the submitted work other than that described above; no financial relationships with any organizations that might have an interest in the submitted work; no other relationships or activities that could appear to have influenced the submitted work.
